# Study on the current situation and spatial distribution of intimate partner violence among Chinese residents

**DOI:** 10.3389/fpubh.2025.1491747

**Published:** 2025-02-20

**Authors:** Ruofan Zhang, Ge Qu, Yuchao Sun, Jing Feng, Zihui Lei, Xinyan Li, Aoqi Shen, Yanli Zuo, Yong Gan

**Affiliations:** ^1^Department of Social Medicine and Health Management, School of Public Health, Tongji Medical College, Huazhong University of Science and Technology, Wuhan, China; ^2^School of General Practice, Guangxi Medical University, Nanning, China

**Keywords:** intimate partner violence, spatial distribution, GIS, spatial autocorrelation, hotspot analysis

## Abstract

**Background:**

Intimate partner violence (IPV), defined as any behavior within an intimate relationship that causes physical, psychological, or sexual harm to those in the relationship, is a significant public health issue worldwide. To analyze the current status and spatial distribution patterns of IPV among residents in China, and identify the nationwide prevalence trends and regions of high severity, so as to provide a scientific basis for the formulation and implementation of government interventions.

**Methods:**

A multi-stage sampling approach was employed to conduct a psychological and behavioral survey among 31,449 residents in 148 cities across the nation from June to August 2022. IPV was measured using a self-developed scale that was specifically designed and culturally adapted for the Chinese context, and it was been categorized into psychological violence, physical violence and sexual violence. Geographic information system (GIS) technology and spatial analysis methods was applied. GeoDa 1.18, ArcGIS 10.8 and STATA 17 software were utilized for data analysis.

**Results:**

The prevalence rates of IPV among Chinese residents was 45.80%, and the prevalence rates of psychological violence, physical violence and sexual violence was 44.50, 21.65 and 18.96%, respectively. The standard deviation plot of prevalence rates across provinces revealed that residents in Shanghai consistently had higher rates of all three categories of IPV compared to the national average level. The results of local spatial auto-correlation analysis indicated that there was a high-high clustering pattern of overall prevalence rates of IPV in Jiangxi and Zhejiang provinces, and a high-low clustering pattern was observed in Jilin, Hebei, and Ningxia provinces. The distribution pattern of intimate partner psychological violence prevalence rates showed a similar clustering pattern as the overall IPV. Additionally, there was a low-low clustering pattern of intimate partner physical violence in Anhui province, and a low-low clustering pattern of intimate partner sexual violence was identified in Anhui and Shandong provinces.

**Conclusion:**

The prevalence rates of IPV in China was relatively high, especially in Shanghai, and there was a certain degree of spatial distribution difference, which urgently needs to be paid attention to by relevant departments and institutions, especially around Zhejiang and Jiangxi in eastern China.

## 1 Introduction

The World Health Organization (WHO) defined Intimate Partner Violence (IPV) as “physical, psychological, or sexual violence committed or threatened by a current or former partner” (1), which encompasses not only domestic violence traditionally associated with married couples but also violence occurring between cohabiting opposite-sex partners, same-sex partners, and individuals in other intimate relationships (2). Currently, IPV had emerged as a significant global public health concern (3), studies conducted both domestically and internationally have shed light on the high prevalence rates of IPV among partners. In 2021, the WHO had released the report *Violence Against Women prevalence Estimates (Intimate Partner Violence and Non-Partner Sexual Violence), 2018*, based on data from 2010 to 2018. It was estimated that in 2018, the lifetime prevalence of IPV among married or partnered women aged 15 years and older was 26%, while the past-year prevalence was 10%. Women aged 20 to 44 showed the highest lifetime prevalence, ranging from 26 to 28%. The report highlighted the widespread nature of IPV perpetrated by male partners and the significant risks faced by younger women (4).

IPV was recognized as a significant contributing factor to depression, infertility, and even suicides (5, 6). Furthermore, IPV has negative impacts on children, leading to issues such as anxiety, depression, sleep disorders, and aggression (7), children who witnessed IPV between their parents were more likely to become perpetrators of violence themselves (8). At a societal level, IPV had imposed a substantial economic burden. Peterson et al. (9) revealed that IPV imposed a total economic burden of 3.6 trillion dollars (in 2014) on the adult population (>18 years old) in the United States, with the governments should bearing 37% (1.3 trillion). The study by Brown et al. (10) showed that the annual health burden was associated with physical IPV among females aged 13 to 24 in Colombia amounts to 90.6 million USD. France spent up to 247.2 billion euros annually on public expenses related to domestic violence (11). These results underscored the substantial consumption of public resources driven by IPV and highlighted the significant strain it places on societal and public financial systems. IPV not only affects individual well-being and happiness but also has significant negative implications for social economy and culture. Thus, urgent attention and intervention are required to address the IPV situation in China.

Characterizing the spatial distribution of public health is a vital undertaking in epidemiology, given that around 80% of epidemiological research data possess spatial attributes (12). Currently, spatial distribution analysis based on Geographic Information Systems (GIS) had been widely employed in exploring the etiology and intervention measures for public health issues such as infectious diseases and chronic conditions (13–15). Previous studies had indicated that IPV was influenced by various factors, including social and cultural factors, educational levels, income disparities between partners, and personal experiences (16–18), given the vast territory of China and the significant differences in economic development and cultural beliefs across regions (19, 20), the prevalence rates of IPV may vary considerably in different areas. However, to date, no study has analyzed the spatial variations of IPV among Chinese residents.

This study aimed to systematically analyze the current status and spatial distribution patterns of IPV among residents in China using GIS technology, providing a comprehensive understanding of national trends and identifying high-risk areas. By constructing high-risk area maps, this study offers scientific evidence to optimize the allocation of public resources, ensuring that anti-violence resources—such as financial support, institutional infrastructure, and legal services—will be prioritized in the most severely affected regions. For regions with high prevalence rates of IPV, this study emphasizes the need to enhance relevant legal frameworks and public service systems. Conversely, in regions with low prevalence rates of IPV, the study seeks to summarize regional characteristics, investigate the underlying factors contributing to the low prevalence, and effectively promote these strategies to high prevalence rates areas, thereby establishing a more targeted and integrated approach to IPV prevention and intervention.

## 2 Methods

### 2.1 Study population and sampling

The data for this study was derived from the Psychology and Behavior Investigation of Chinese Residents (PBICR), conducted from June 20th to August 31st, 2022. A multi-stage sampling approach was employed across all provincial-level administrative regions in China (excluding Hong Kong, Macau, and Taiwan), encompassing a total of 148 cities. The sampling was stratified at the levels of province, city, district/county, township/street, and community/village. Quota sampling was employed at the community/village to individual level. A total of 31,449 questionnaires were collected. Each province, municipality, or autonomous region was assigned a designated surveyor, while each city recruited at least one surveyor or survey team. The surveyors established questionnaire survey points at the local health service centers or relevant health service stations within their assigned communities. Recruitment notices were distributed to invite participants, and their identities were subsequently verified to ensure compliance with the inclusion and exclusion criteria of the study. During the survey period, surveyors were required to administer electronic questionnaires to participants through one-on-one, on-site (if conditions permitted), or videoconferencing (due to restrictions imposed by the COVID-19 pandemic). The electronic questionnaires were created by using the Wenjuanxing platform.^1^ After obtaining informed consent from the participants, the surveyors either input the questionnaire number themselves or informed the participants of the questionnaire number for them to complete the survey. In cases where participants were cognitively capable but unable to answer the questionnaire independently, the surveyors conducted detailed interviews and recorded their responses separately. The inclusion criteria for the survey participants were as follows: (1) Chinese citizens of the People’s Republic of China, (2) Chinese residents (with no more than 1 month spent outside of China in a year), (3) voluntary participation in the study, with the completion of an informed consent form, (4) ability to independently complete the online questionnaire or with the assistance provided by the surveyors, and (5) understanding the meaning of each item in the questionnaire.

This study has received ethical approval from the Institutional Review Board of Shaanxi International Trade Business College (No. JKWH-2022-02).

### 2.2 Prior survey

Prior to the formal survey, this study conducted three rounds of pilot surveys from June 5 to June 8, June 10 to June 13, and June 15 to June 18, 2022. The sampling method used in the pilot surveys was quota sampling, with the quota attributes aligned with those of the formal survey. The sample sizes for the three rounds were 100, 100, and 200 participants, respectively. During the pilot surveys, feedback from respondents was collected and systematically organized. The reliability of the questionnaire was evaluated through statistical analysis, and revisions were made based on discussions among the research team members. The revised version of the questionnaire was then resubmitted for expert review. The final questionnaire was finalized after incorporating modifications from the three rounds of pilot surveys. It is important to note that data collected during the pilot phase were excluded from the final research analysis (21).

### 2.3 Measurement

In this study, a culturally adapted self-developed IPV scale was utilized, which was designed according to the Chinese context by referring Straus et al.’s (22) revised Conflict Tactics Scale was utilized. This scale primarily assessed the experiences of IPV among victims, encompassing three dimensions: psychological violence, physical violence, and sexual violence, with a total of five items. One item assessed physical violence, including direct physical assault or harm inflicted using objects. One item evaluated sexual violence, encompassing unwanted physical contact or sexual acts against one’s will. Three items measured psychological violence, including neglect (lack of concern during periods of physical discomfort or emotional distress), control (monitoring of phone usage, control over dressing and appearance, and restrictions on social interactions), and emotional violence (comparison with others, public accusations, leading to feelings of embarrassment and diminished self-confidence). The scale employed a 5-point Likert to assess the frequency of IPV experiences (never, rarely, sometimes, often, almost always).In this study, the Cronbach’s ɑ coefficients for the total IPV scale and the psychological violence subscale were 0.91 and 0.86, respectively.

For any item on the scale, a response other than “never” indicated the presence of IPV experiences. If the response to the physical violence item was anything other than “never,” it was considered as experiencing physical violence. Similarly, if the response to the sexual violence item was anything other than “never,” it was considered as experiencing sexual violence. For the three items measuring psychological violence, if any item had a response other than “never,” it was considered as experiencing psychological violence.

### 2.4 Spatial auto-correlation analysis

#### 2.4.1 Moran’s index

The analysis included univariate Global Moran’s index and Anselin Local Moran’s index. By applying queen weights to the pre-existing map dataset, this study examined the spatial clustering of IPV among residents in different regions. The global Moran’s index ranges from −1 to 1, with the absolute value indicating the degree of spatial autocorrelation. The closer the absolute value was to 1, the stronger the spatial correlation. The positive/negative sign represented the positive/negative direction of the correlation, while 0 indicated no correlation (23). The calculation formula for the Moran’s I statistic is as follows (Equation 1) (24):


(1) $$I=\frac{n\sum_{i=1}^{n}\sum_{j=1}^{n}w_{ij}z_{i}z_{j}}{S_{0}\sum_{i=1}^{n}Z_{i}^{2}}$$

Where *Z*_i_ is the deviation of an attribute for feature *i* from its mean (*x_i_*-*X*), *w_ij_* is the spatial weight between *i* and *j*, *n* is equal to the total number of its features and *S_0_* is the aggregate of all the spatial weights (Equation 2).


(2) $$S_{0}=\overset{n}{\underset{i=1}{\sum }}\overset{n}{\underset{j=1}{\sum }}w_{ij}$$

LISA cluster analysis classifies clustering patterns into four types: high-high clustering pattern means that areas with high levels of IPV were positively correlated with neighboring provinces with high levels, low-low clustering pattern means that areas with low levels of IPV were positively correlated with neighboring provinces with low levels, high-low clustering pattern means that areas with high levels of IPV were negatively correlated with neighboring provinces with low levels, and low-high clustering pattern means that areas with low levels of IPV were positively correlated with neighboring provinces with high levels (25).

#### 2.4.2 Hotspot analysis

This study used the Getis-Ord Gi* statistic to analyze the spatial distribution of hotspots and coldspots within the study area (26). This method was used to evaluate the spatial association between the attribute values of a spatial unit and those of its neighboring units, identifying significant hotspots (clusters of high values) and coldspots (clusters of low values). A spatial weight matrix based on the queen weights was used to construct the adjacency matrix. The calculation formula for the Gi* statistic is as follows (Equation 3) (24):


(3) $$G_{i}^{*}=\frac{\sum_{j=1}^{n}w_{ij}x_{j}-\bar{X}\sum_{j=1}^{n}w_{ij}}{S\sqrt{\frac{\sum_{j=1}^{n}w_{ij}^{2}-{\left(\sum_{j=1}^{n}w_{ij}\right)}^{2}}{n-1}}}$$

Where G^*^_i_ represents the Getis-Ord *Gi* statistic for unit *i*, *x_j_* donate the IPV prevalence of unit *j*, *w_ij_* represents the spatial weight between units *i* and *j*, and $\bar{X}$ and *S* are the mean and standard deviation of the IPV prevalence, respectively.

### 2.5 Statistical analysis

Data filtering, calculation, and transformation were conducted using Stata 17.0 software. The calculation method for the provincial-level prevalence rates of IPV was as follows: First, data on each respondent’s experience with IPV was collected through survey instruments. Next, the data was aggregated within each provincial administrative region, calculating the total number of individuals who had experienced IPV. This number was then divided by the total number of respondents in the region to obtain the prevalence rates of IPV and its various dimensions at the provincial level. The processed data was merged with the administrative area vector map of China, obtained from the Alibaba Cloud DataV-Data Visualization Platform,^2^ using GeoDa 1.18 and ArcGIS 10.8.2 software, followed by assigning spatial weights and conducting spatial autocorrelation analysis.

## 3 Results

After data cleaning, a total of 30,505 valid questionnaires were collected in this study. Among them, there were 13,229 male participants (43.37%) and 17,276 female participants (56.63%). The age of the surveyed individuals was mainly concentrated between 12 and 59 years, with 26,154 cases (85.74%). Regarding educational background, the majority of the surveyed individuals had completed a junior college and above degree, totaling 15,578 cases (51.07%). The predominant family type was nuclear families (consisting of parents and unmarried children), with 15,885 cases (52.80%) (Table 1).

**Table 1:** Demographic characteristic of study population.

Characteristic	Frequency	Proportion
Gender		
Male	13,229	43.47%
Female	17,276	56.63%
Educational level		
Primary school and below	7,726	25.33%
High school and vocational School	7,201	23.60%
Junior college and above	15,578	51.07%
Location		
Northern region	6,277	20.58%
Northeast region	1835	6.02%
Eastern region	6,049	19.83%
Central region	2,771	9.08%
Southern region	4,006	13.13%
Southwest region	4,825	15.82%
Northwest region	4,742	15.54%
Age (years)		
12 ~ 59	26,154	85.74%
>60	4,351	14.26%
Family types		
Married couple	6,164	20.21%
Core family	15,885	52.07%
Nuclear family	4,563	14.96%
Skip-generation family	229	0.75%
Blended family	817	2.68%
Single-parent family	1,170	3.84%
Single-person household	710	2.33%
DINK (Double income, no kids) family	245	0.80%
Other forms of family	722	2.37%

“DINK” is an acronym that stands for “Dual Income, No Kids.” It refers to a household in which both partners are earning an income and have chosen not to have children.

### 3.1 Prevalence of IPV among Chinese residents

Among the 30,505 cases surveyed, there were 13,970 cases (45.80%) exposure to IPV, with 6,604 cases (21.65%) of physical violence, 5,784 cases (18.69%) of sexual violence, and 13,574 cases (44.50%) of psychological violence.

Gender-stratified analysis showed that 6,158 cases (46.55%) of IPV were reported by males, and 7,812 cases (45.22%) were reported by females. Among males, there were 3,243 cases (24.51%) of physical violence, 2,900 cases (21.92%) of sexual violence, and 5,992 cases (45.29%) of psychological violence. Among females, there were 3,361 cases (19.45%) of physical violence, 2,884 cases (16.69%) of sexual violence, and 7,582 cases (43.89%) of psychological violence. Chi-square tests revealed significant differences in the prevalence rates of IPV and its three dimensions among residents of different genders (*p*<0.05) (Table 2).

**Table 2:** Gender-stratified analysis of IPV among Chinese residents.

Items	Gender		*χ^2^*	*p*
	Male	Female		
IPV	6,158 (46.55%)	7,812 (45.22%)	5.34	0.021
Physical violence	3,243 (24.51%)	3,361 (19.45%)	113.07	<0.01
Sexual violence	2,900 (21.92%)	2,884 (16.69%)	113.26	<0.01
Psychological violence	5,992 (45.29%)	7,582 (43.89%)	6.01	0.014

### 3.2 Spatial statistical analysis

The prevalence rates of IPV in various provincial-level administrative units in China range from 33.24 to 64.44%, with higher rates observed in Shanghai, Jilin, Hunan, Ningxia, and the Tibet Autonomous Region. The range of prevalence rates for physical violence was from 12.50 to 42.70%, with higher rates observed in Shanghai, the Tibet Autonomous Region, Liaoning, Tianjin, and Jilin. The prevalence rates of sexual violence range from 11.93 to 39.02%, with higher rates observed in Shanghai, the Tibet Autonomous Region, Liaoning, Tianjin, and Jilin. Psychological violence exhibits prevalence rates ranging from 31.49 to 61.54%, with the same provinces having higher rates as those observed for physical violence (Table 3).

**Table 3:** Prevalence of IPV and its three dimensions in China.

Province	IPV	Physical violence	Sexual violence	Psychological violence
Northern region	40.79%	20.61%	18.83%	39.68%
Beijing	39.31%	17.35%	15.93%	38.57%
Tianjin	52.51%	29.15%	25.88%	51.01%
Inner Mongolia autonomous Region	33.24%	20.11%	18.89%	31.49%
Hebei	48.55%	22.38%	20.10%	47.91%
Shanxi	45.05%	22.14%	19.27%	44.27%
Northeast region	47.32%	27.49%	25.48%	46.36%
Heilongjiang	36.79%	16.56%	14.05%	36.45%
Jilin	53.89%	27.63%	26.07%	52.53%
Liaoning	48.02%	32.29%	30.74%	46.74%
Eastern region	50.21%	21.87%	18.15%	48.65%
Shanghai	64.44%	42.70%	39.02%	61.54%
Shandong	40.94%	18.12%	16.17%	39.91%
Anhui	51.17%	19.79%	14.96%	49.19%
Zhejiang	51.61%	20.88%	18.34%	50.93%
Jiangxi	49.02%	16.44%	11.93%	47.72%
Fujian	52.04%	22.45%	17.35%	51.02%
Jiangsu	40.99%	16.54%	14.15%	40.07%
Central region	46.26%	19.56%	17.39%	45.25%
Henan	42.96%	15.91%	13.21%	42.22%
Hubei	49.19%	26.21%	23.95%	47.57%
Hunan	55.83%	25.00%	25.24%	54.61%
Southern region	43.78%	21.19%	17.15%	42.21%
Guangxi	36.89%	16.52%	14.81%	35.73%
Guangdong	51.09%	21.38%	15.17%	49.96%
Hainan	41.98%	24.57%	20.82%	40.20%
Southwest region	47.98%	22.74%	19.96%	46.82%
Tibet autonomous region	52.68%	34.59%	34.19%	51.69%
Yunnan	49.46%	20.84%	19.04%	48.38%
Sichuan	47.12%	20.95%	17.52%	45.99%
Chongqing	46.32%	23.53%	18.63%	45.34%
Guizhou	46.80%	21.89%	17.93%	45.29%
Northwest region	45.36%	20.94%	19.21%	43.76%
Xinjiang Uygur autonomous Region	43.59%	16.35%	14.74%	42.63%
Qinghai	48.13%	19.54%	15.66%	47.13%
Gansu	41.26%	14.57%	14.25%	39.37%
Ningxia Hui autonomous Region	52.94%	25.42%	24.16%	52.10%
Shaanxi	45.82%	26.79%	24.70%	43.74%

A Global Moran’s I analysis was conducted on the overall IPV prevalence rates and its three dimensions in China, which revealed no significant spatial clustering relationships.

**Figure 1:**
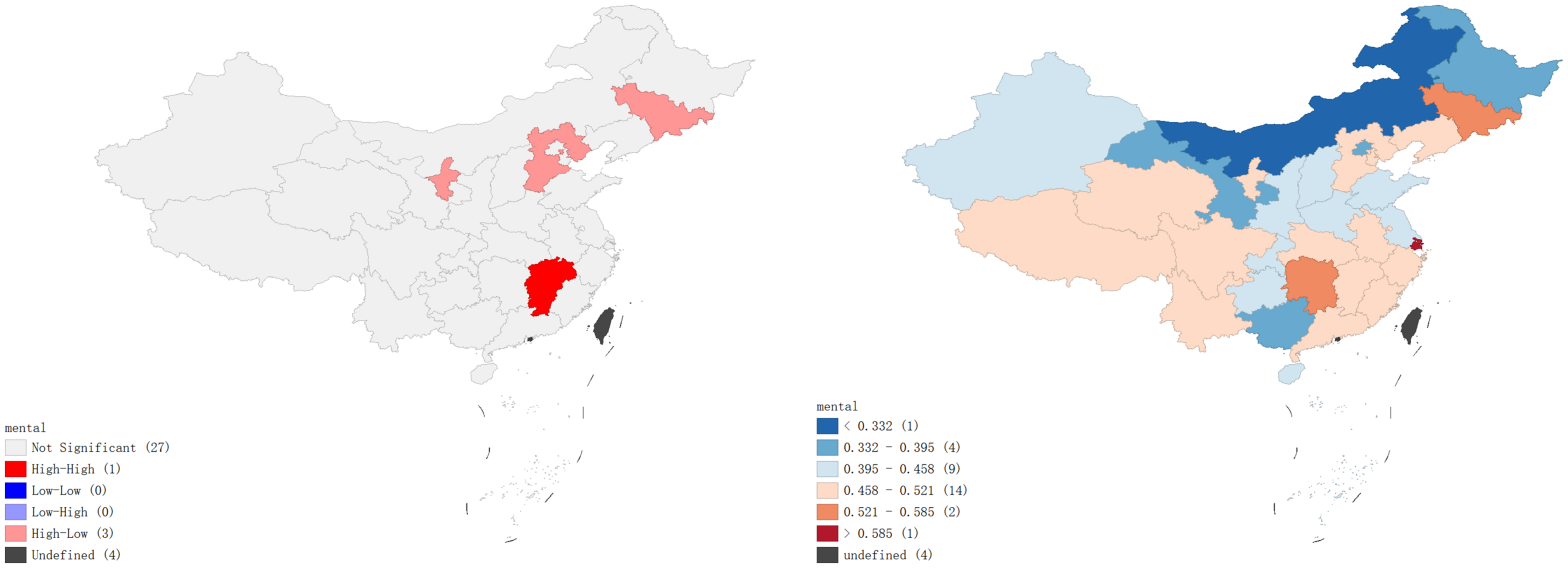
Overall prevalence rates of violence – Lisa clustering map and standard deviation graph.

**Figure 2:**
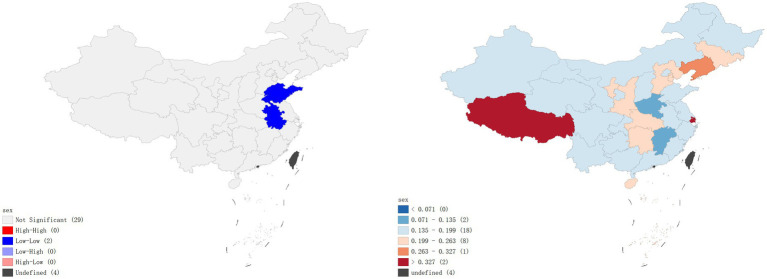
Prevalence rates of physical violence – Lisa clustering map and standard deviation graph.

**Figure 3:**
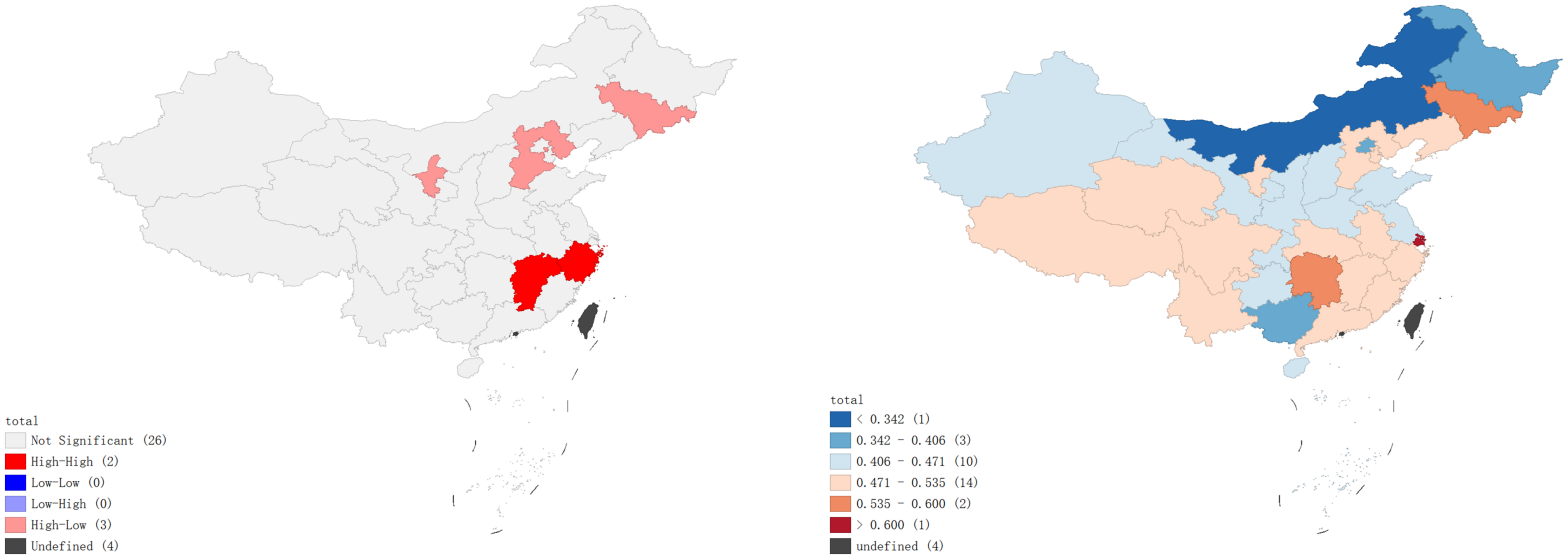
Prevalence rates of psychological violence – Lisa clustering map and standard deviation graph.

**Figure 4:**
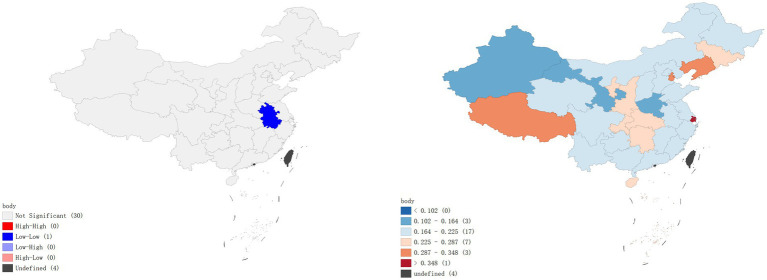
Prevalence rates of sexual violence – Lisa clustering map and standard deviation graph.

A local Moran’s I analysis of IPV prevalence rates in China identified provinces with a high-low spatial clustering pattern, including Jilin Province, Ningxia Hui Autonomous Region, and Hebei Province, which were adjacent to Inner Mongolia Autonomous Region, Gansu Province, Shaanxi Province, and Heilongjiang Province, all with low prevalence rates of IPV. Provinces with high-high spatial clustering pattern included Jiangxi Province and Zhejiang Province (*p* < 0.01), which were adjacent to Hunan Province, Fujian Province, Anhui Province, and Guangdong Province, all with high prevalence rates of IPV (Figure 1). In terms of physical violence, there exists a low-low spatial clustering pattern in Anhui province (Figure 2). In the dimension of psychological violence, the results of spatial auto-correlation analysis were consistent with the local Moran’s I analysis of overall IPV prevalence rates (Figure 3). In the dimension of sexual violence, Shandong Province and Anhui Province exhibited a low-low spatial clustering relationship, adjacent to Anhui Province and Jiangsu Province, both with a lower prevalence rates of IPV (Figure 4).

**Figure 5:**
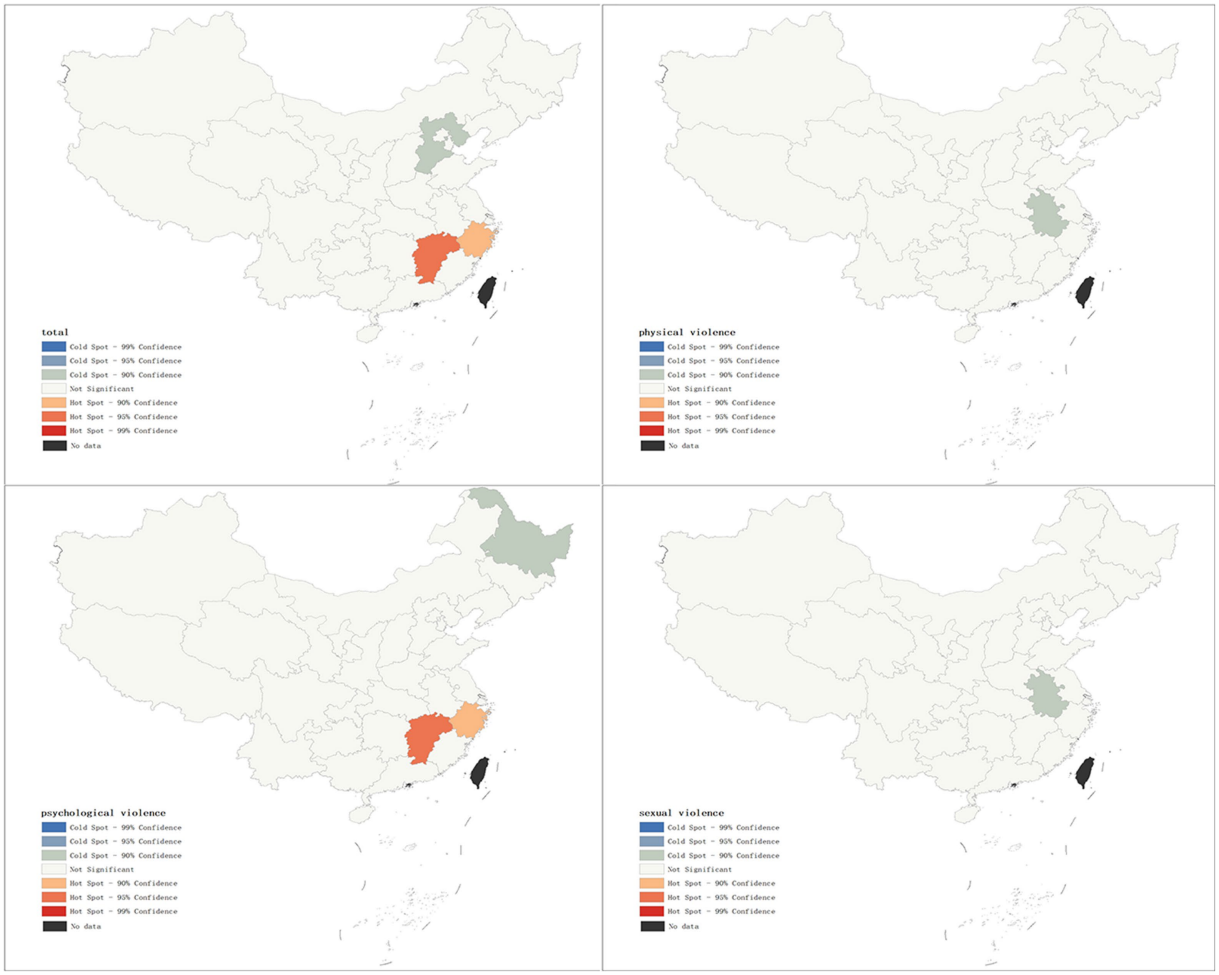
Spatial distribution of hot and cold spots of IPV.

As shown in Figure 5, the results of the hot spot analysis were largely consistent with those of the local spatial autocorrelation analysis. The findings indicated that hotspots of overall IPV prevalence and psychological violence prevalence were primarily located in Jiangxi and Zhejiang provinces, while cold spots were located in Hebei and Heilongjiang provinces, respectively. No significant hotspots were identified for physical violence or sexual violence, but cold spots were found in Anhui province.

## 4 Discussion

This study revealed an average IPV prevalence rates of 45.80% in China, with rates of psychological violence, physical violence, and sexual violence at 44.50, 21.65, and 18.96%, respectively. Compared to both domestic and international studies, China’s overall IPV prevalence rates was moderately high on a global scale (3). In comparison to data released by the U.S. CDC in 2018, the prevalence rates of IPV among women in China was 8.82% higher, while among men, it was 12.95% higher (27). Similarly, when compared to data from the UK in 2014, the IPV prevalence in China is 17.42% higher for women and 27.85% higher for men (28). In the contrast, compared to a study on IPV among women conducted in Uganda, the overall IPV prevalence was 10.2% lower. Specifically, the prevalence rates of physical violence, sexual violence, were 19.35, 4.31% lower, while the prevalence of psychological violence was 4.5 higher.

Stratified analysis of IPV prevalence rates among different genders and dimensions showed that males experience IPV at a higher frequency than females. This finding challenges traditional beliefs that females, as the vulnerable party, were more likely to be victims of IPV. Previous research has generally focused on the higher probability of female IPV victimization, as seen in the survey results of Li (29) and Joksimovic et al. (30). Several factors may contribute to this situation. Firstly, with the rapid development of the internet and the diversification of employment opportunities, the employment landscape had expanded, including new job formats. As a result, women had gradually broadened their employment prospects and gained a certain level of economic status within their households, thereby reducing the risk of IPV (31). Secondly, lower levels of education were also an important risk factor influencing IPV prevalence rates, Silva’s study (32) demonstrated that women who have received 4 years or less of schooling were 4.5 times more likely to experience physical violence compared to those with five or more years of education. In recent years, the comprehensive implementation of compulsory education policies in China has significantly elevated the overall educational level of the population, thereby reducing the risk of IPV for women. Furthermore, China has actively promoted legislation and law enforcement efforts against male domestic violence. The implementation of the “Anti-Domestic Violence Law” in 2016 has taken China’s efforts to combat domestic violence to a new level (33). This legislation primarily focuses on protecting women’s rights and emphasizes the protection of women, thus reducing the likelihood of female IPV at a legal level.

### 4.1 Prevalence of IPV among residents in China exhibits significant spatial distribution differences

The results of local Moran’s analysis indicated that in Eastern China, the prevalence rates of total IPV and t psychological violence showed high-high clusters, whereas physical violence and sexual violence exhibited low-low cluster. The underlying reason for this phenomenon warrant further investigation. As an economically developed region, East China ranked among the top in the nation in terms of socioeconomic indicator. According to the *2023 China Statistical Yearbook* (34), the region’s gross domestic product (GDP) reached 4.6 trillion RMB, and its expenditure on education amounted to 1.8 trillion RMB, both the highest in China. Moreover, the proportion of residents with a college degree or above stood at 21.31%, the second highest in China. These factors suggest that East China’s economic prosperity not only ensure abundant educational resources but also attracts highly skilled talents from across the country, resulting in a relatively high overall level of education among its resident. Thus, conflicts within intimate relationships may be less likely to escalate into physical confrontations and be more likely to manifest as emotional harm. Additionally, higher levels of education may heighten individuals’ sensitivity to emotional distress (35).

High-low clustering of IPV and the psychological violence dimension of IPV is observed in Jilin Province, Hebei Province, and the Ningxia Hui Autonomous Region. Previous research by Zhao et al. (36) has reported a high prevalence of IPV in rural areas of Jilin Province. The study attributed the higher IPV rates in rural Jilin Province compared to Chongqing Municipality and Anhui Province to local traditions, customs, low awareness among women regarding IPV, and misconceptions about IPV. Similarly, Gao and Tamara (37) provided a similar explanation for the high prevalence of IPV in a county in Ningxia.

### 4.2 Tailoring IPV prevention and control strategies to local conditions

This study revealed significant regional differences in the prevalence of IPV in different parts of China. Overall, the IPV occurrence was relatively low in the northwest and southwest regions, moderate in the north and northeast regions, and most severe in the southeastern region. For the prevention and intervention of IPV, region-specific measures tailored to local characteristics should be adopted to effectively address these disparities.

For the high prevalence of IPV in the Southeast region, interventions should focus on the following aspects: First, individuals with higher educational attainment tend to exhibit greater sensitivity to emotional harm. However, the current education system pays insufficient attention to emotional education and mental health training. Therefore, given the demographic characteristics of southeastern residents, emotional education and mental health support mechanisms should be introduced. Emotion-focused education programs targeting different age groups should be implemented in schools and communities to develop residents’ skills in emotional expression and conflict resolution within intimate relationships, thereby mitigating the psychological violence stemming from emotional conflicts. Second, the hidden and persistent nature of psychological violence poses unique challenges for its identification and intervention. Therefore, specialized tools for screening psychological violence should be developed to facilitate targeted monitoring, assessment, intervention, and management. Such tools can assist government agencies and social service organizations in formulating integrated intervention strategies. At present, there is no screening tool specifically designed for psychological violence among the general population in China. Yan et al. (38) developed a preliminary Family Cold Violence Scale, which demonstrated good reliability and validity in assessing cold violence within marriage among wives of gays. This study provided a valuable reference for developing screening tools for psychological violence in the general population. Future efforts should expand its applicability and enhance its effectiveness in evaluating psychological violence.

For the high prevalence of IPV in Jilin Province and Ningxia Province, efforts should start with raising awareness and knowledge among women about IPV, aiming to reduce the negative impact of IPV on women, Tiwari et al. (39) categorized IPV-affected Chinese women into abused pregnant women, women in shelters, community-dwelling abused women, and abused mainland immigrant women, and implemented targeted publicity interventions for each group. The research results showed significant effects of targeted publicity interventions in alleviating psychological problems and physical symptoms caused by IPV in women. Additionally, promoting legal education is essential to enhance women’s legal awareness, enabling them to understand and utilize the law to protect their rights effectively. At the same time, attention must be paid to the implementation of relevant laws in judicial practice. Empirical research by Zeng (40) highlighted several challenges women face when applying for protection orders after experiencing IPV, including inappropriate evaluation criteria, difficulties in identifying non-physical forms of violence, negative outcomes from police intervention, and the absence of functional support from women’s federations. These findings suggest that, beyond raising victims’ legal awareness, it is imperative to strengthen the professional knowledge of judicial personnel, particularly through training on issues related to psychological violence, sexual violence, and economic control, to improve the effectiveness of law enforcement. Finally, addressing IPV requires a comprehensive social systems approach, involving multiple public service sectors and social channels. Currently, China lacks a systematic framework with clear procedures for addressing IPV, and the integration of inter-agency mechanisms remains a significant challenge. Therefore, it is necessary to designate a specific government agency as the lead entity, providing detailed regulations on intervention procedures for IPV incidents and clearly defining the roles and responsibilities of relevant departments, as well as their collaboration processes. This approach would facilitate the development of a coordinated, multi-agency response system to effectively address IPV.

### 4.3 Strengths and limitations

The strength of this study lay in its collection of a nationwide large-scale IPV survey, with a sample size of 30,505 and nationwide coverage, providing a high level of representativeness. Moreover, this study represented a pioneering utilization of GIS-based spatial distribution analysis to comprehensively investigate disparities in prevalence rates and spatial clustering patterns of IPV across diverse provinces in China, which was of great value in exploring the influencing factors and prevention measures of IPV.

However, there were still limitations to this study. On one hand, there might be significant differences in IPV situations within different regions of the same province. In this survey, data processing was conducted on a provincial level, without considering the regional differences within provinces. Therefore, further refinement of the regional division can be done in future studies. On the other hand, IPV victims might not recognized the behaviors they experienced as violence mentioned in the questionnaire, or they might have considered it disgraceful to disclose the presence of IPV in their families, or they might have underreported or concealed IPV due to psychological or physical threats from the perpetrators, leading to potential information bias in the survey results and underestimation of the prevalence of IPV (39). Therefore, in future research, data processing units can be further refined, with the minimum unit set as the municipal level, and by comparing data within and outside provinces, more characteristics of IPV prevalence and distribution may be discovered.

In conclusion, IPV in China was relatively severe, ranked above the global average, and exhibited significant gender differences and spatial distribution disparities influenced by various factors such as economy and education. Therefore, efforts should be made to address the causes of distribution disparities and implement tailored IPV prevention and control measures, aiming to reduce the prevalence of IPV, enhance residents’ sense of security and happiness, and ensure their quality of life and physical and mental well-being.

## Data Availability

The raw data supporting the conclusions of this article will be made available by the corresponding authors, upon reasonable request.

## Footnotes

^1^ https://www.wjx.cn/

^2^ datav.aliyun.com
